# Bioactive Fraction of Geopropolis from *Melipona scutellaris* Decreases Neutrophils Migration in the Inflammatory Process: Involvement of Nitric Oxide Pathway

**DOI:** 10.1155/2013/907041

**Published:** 2013-04-30

**Authors:** Marcelo Franchin, Marcos Guilherme da Cunha, Carina Denny, Marcelo Henrique Napimoga, Thiago Mattar Cunha, Bruno Bueno-Silva, Severino Matias de Alencar, Masaharu Ikegaki, Pedro Luiz Rosalen

**Affiliations:** ^1^Department of Physiological Sciences, School of Dentistry of Piracicaba, University of Campinas, 901 Limeira Ave., 13414 903 Piracicaba, SP, Brazil; ^2^Laboratory of Immunology and Molecular Biology, São Leopoldo Mandic Institute and Research Center, 13 José Rocha Junqueira Ave., 13045-755 Campinas, SP, Brazil; ^3^Department of Pharmacology, School of Medicine of Ribeirão Preto, University of São Paulo, 3900 Bandeirantes Ave., 14049-900 Ribeirão Preto, SP, Brazil; ^4^Department of Agri-Food industry, Food and Nutrition, “Luiz de Queiroz” College of Agriculture, University of São Paulo, 11 Pádua Dias Ave., 13418-900 Piracicaba, SP, Brazil; ^5^School of Pharmaceutical Sciences, Federal University of Alfenas, 700 Gabriel Monteiro da Silva St., 37130-000 Alfenas, MG, Brazil

## Abstract

The aim of this study was to evaluate the activity of the ethanolic extract of geopropolis (EEGP) from *Melipona scutellaris* and its fractions on the modulation of neutrophil migration in the inflammatory process, and the participation of nitric oxide (NO) pathway, as well as to check the chemical profile of the bioactive fraction. EEGP and its aqueous fraction decreased neutrophil migration in the peritoneal cavity and also the interaction of leukocytes (rolling and adhesion) with endothelial cells. The levels of chemokines CXCL1/KC and CXCL2/MIP-2 were not altered after treatment with EEGP and the aqueous fraction. It was found that the injection of NO pathway antagonists abolished the EEGP and the aqueous fraction inhibitory activity on the neutrophil migration. The expression of intercellular adhesion molecule type 1 (ICAM-1) was reduced, and nitrite levels increased after treatment with EEGP and aqueous fraction. In the carrageenan-induced paw edema model, EEGP and the aqueous fraction showed antiedema activity. No pattern of flavonoid and phenolic acid commonly found in propolis samples of *Apis mellifera* could be detected in the aqueous fraction samples. These data indicate that the aqueous fraction found has promising bioactive substances with anti-inflammatory activity.

## 1. Introduction

Neutrophils have key involvement in defending the body during the inflammatory process. The process of neutrophil rolling and adhesion to endothelial cells, followed by its transmigration to the extravascular space, occurs due to the release of lipidic mediators, cytokines and chemokines, which activate selectins, integrins, and immunoglobulin [1, 2].

On the other hand, although recruitment of neutrophils is a protective response of the organism, the occurrence of an intense response can produce undesirable effects, that can lead to a progressive tissue damage in the inflamed site. This phenomenon occurs in different inflammatory diseases, such as rheumatoid arthritis and periodontal disease [3, 4].

The development of new anti-inflammatory drugs that interfere with the neutrophils trafficking on the inflammatory focus has attracted great interest among researchers, and the sought strategies are by inhibiting of rolling, transmigration, and adhesion of neutrophils on the inflammatory focus, either by blocking of molecules involved in this process or stimulating mediators such as nitric oxide (NO), which inhibits this event [5–7].

Natural products have been researched for decades as a promising source in the discovery of new drugs, and *Apis mellifera* bee propolis has been reported in the literature as possessing various biological activities [8–10]. In addition, *Apis mellifera *propolis has been demonstrated as a source of natural resource for the discovery of new bioactive compounds, such as artepelim C [11], apigenin and *tt*-farnesol [12], and CAPE [9], among others.

Geopropolis, a mixture of resin, wax, and soil, is an uncommon propolis collected by native stingless bees of the Meliponini tribe and widely found in tropical and subtropical areas worldwide [13, 14]. Among the geopropolis, the one from the *Melipona scutellaris* bee species has been target of interest of our research group. Studies with geopropolis observed significant antimicrobial activity against *Staphylococcus aureus* [15] and antinociceptive activity [16], suggesting that further studies should be conducted in order to identify other biological activities as well as the elucidation of its whole chemical profile, aimed at identifying promising chemicals with pharmacological potential.

Thus, the aim of this study was to evaluate the activity of the ethanolic extract of geopropolis (EEGP) from *Melipona scutellaris* and its fractions on the modulation of neutrophil migration in the inflammatory process, and the participation of NO pathway, as well as to check the chemical profile of the bioactive fraction.

## 2. Material and Methods

### 2.1. Geopropolis Samples and Fractionation

The geopropolis samples were collected between June and July 2010 in the seaside region, municipality of Entre Rios (11°57′ S, 38°05′ W), state of Bahia, Northeast of Brazil. Geopropolis (100 g) was extracted with absolute ethanol (w/v) of proportion (1/7), at 70°C, for 30 min, and then filtered to obtain the EEGP. The EEGP was further fractioned using a liquid-liquid extraction technique with hexane, chloroform, and ethyl acetate solvents. At the end of three partitions, it was obtained a residue called aqueous fraction [16]. The fractions obtained were monitored by thin layer chromatography (TLC) using the anisaldehyde reagent, followed by incubation at 100°C for 5 min. Fluorescent substances were visualized under UV light at the wavelengths of 254 and 366 nm [17]. The EEGP and its hexanic, chloroform, ethyl acetate, and aqueous fractions were concentrated in a rotaevaporator at 40°C to obtain yields of 4.33% (w/w), 1.98% (w/w), 0.23% (w/w), 0.87% (w/w), and 1.25% (w/w), respectively. The extract and fractions were dissolved in DMSO 1% (dissolved in PBS at 1 mM) for subcutaneous (s.c.) administration.

### 2.2. Animals

Male SPF (specific-pathogen free) BALB/c mice weighing 20–25 g were housed in temperature of 22–25°C, with a light cycle of 12 h light/12 h dark, humidity of 40–60%, and with access to water and food *ad libitum*. The procedures described were reviewed and approved by the local animal Ethics committee (CEUA Unicamp process no. 2037-1).

### 2.3. Drugs and Reagents

Carrageenan, aminoguanidine (AG), [1H-(1,2,4)oxadiazolo (4,3-a) quinoxalin-1-one] (ODQ), and dimethylsulphoxide (DMSO) were obtained from Sigma Chemical Co. (St. Louis, MO, USA) and organic solvents from Merck.

### 2.4. Biological Protocols

#### 2.4.1. Evaluation of the Activity of EEGP and Its Fraction on Neutrophil Migration in the Peritoneal Cavity Induced by Carrageenan

The mice were pretreated with EEGP, hexane, chloroform, ethyl acetate, or aqueous fractions (1, 3, 10, or 30 mg/kg, s.c.). The negative control group received the vehicle. After 30 min of treatment, it was applied carrageenan IP at a dose of 500 *μ*g/cavity. After 4 h, the animals were killed and the peritoneal cavity washed with 3 mL of PBS/EDTA (1 mM). For total cell counting, it was utilized the Neubauer chamber, and for differential counting, it was performed by preparing smears in a cytocentrifuge (citospin; Shandon Lipshaw Inc., Pittsburgh, PA, USA), which were stained with fast Panotic kit, and for differentiated cells (100 cells total), an optical microscope (1000× increase) was utilized. The results were expressed as number of neutrophils per cavity [18].

#### 2.4.2. Evaluation of the Activity of EEGP and Its Bioactive Fraction on the Rolling and Adhesion of Leukocytes in the Mesenteric Microcirculation by Intravital Microscopy

The mice were pretreated with EEGP or aqueous fraction (10 mg/kg, s.c.) 30 min before the IP injection of carrageenan 500 *μ*g/cavity. The negative control group received the vehicle. After 2 and 4 h since the inflammatory stimulus, the rolling and adhesion of leukocytes were rated as previously described [19, 20]. The animals were anesthetized, and the mesenteric tissue was exposed to *in situ* assessment by intravital microscopy. The animals were placed on a plate with a thermostat at 37°C, where the mesenteric tissue was kept warm and moist with Ringer Locke solution (pH from 7.2 to 7.4) containing 1% of gelatin. The postcapillary venules, which had a diameter of 10–18 *μ*m, were chosen, and the interaction of leukocytes with the luminal surface of the endothelium venule was assessed, where we counted the number of rolling leukocytes for 10 min. Leukocytes were considered adherent to the endothelium if they remained stationary for >30 s. Cells were counted, and the image was recorded using five different fields for each animal to avoid variability due to sampling. Calculations were made for each animal.

#### 2.4.3. Evaluation of Chemokines Levels after Treatment with EEGP and Bioactive Fraction

The mice were pretreated with EEGP or aqueous fraction (10 mg/kg, s.c.). The negative control group received the vehicle. After 30 min of treatment, it was applied carrageenan IP at a dose of 500 *μ*g/cavity. After 3 h, the animals were killed and the peritoneal cavity washed with 3 mL of PBS/EDTA (1 mM). Levels of CXCL1/KC and CXCL2/MIP-2 were determined by ELISA using protocols supplied by the manufacturers (R&D Systems, Inc). The results are expressed as picograms.

#### 2.4.4. Effect of Inhibitors of NO Pathway on the EEGP Inhibitory Effect and Bioactive Fraction on Neutrophil Migration in the Peritoneal Cavity Induced by Carrageenan

The animals were pretreated with a selective inhibitor of iNOS (aminoguanidine 50 mg/kg, s.c.) or a selective inhibitor of soluble guanylate cyclase (ODQ 5 *μ*mol/kg, IP) 30 min before EEGP or aqueous fraction (10 mg/kg, s.c.) administration. The negative control group received the vehicle. After 30 min of treatment, it was applied carrageenan at a dose of 500 *μ*g/cavity and neutrophil migration was determined as described in the Section 2.4.1 [18, 21].

#### 2.4.5. Evaluation of ICAM-1 Expression by Western Blotting after Treatment with EEGP and Bioactive Fraction

The mice were pretreated with EEGP or aqueous fraction (10 mg/kg, s.c.). The negative control group received the vehicle. After 30 min of treatment, it was applied carrageenan IP at a dose of 500 *μ*g/cavity. After 4 h, the animals were killed, then the mesenteric tissue was dissected, and the proteins were isolated. Tissues were lysed in 400 mL of buffer (1% Triton X-100, 1 M NaF, 100 mM Nappi, 1 M Na_3_VO_4_, 1 mg/mL aprotinin, 1 mg/mL leupeptin, and 1 mg/mL PMSF) and centrifuged at 4°C for 20 min at 12.300/g. Equal amounts of protein (50 *μ*g) were separated by 10% SDS-PAGE and transferred to a nitrocellulose membrane (Bio-Rad). The standard molecular weight (Bio-Rad) was run in parallel to estimate the molecular weight. Membranes were blocked, overnight at 4°C, in TBS-T (20 mM Tris-HCl (pH 7.5), 500 mM NaCl, and 0.1% Tween 20) plus 5% of nonfat skimmed milk powder. After the blocking, the membranes were incubated, overnight at 4°C, with rabbit anti-ICAM-1 (1 : 200) or *α*-tubulin (Santa Cruz Biotechnology) and utilized as an internal control (1 : 1000) diluted in TBS-T containing 5% of nonfat skim milk powder. The membranes were then incubated with rabbit anti-IgG conjugated to peroxidase (1/2000) diluted in TBS-T containing 5% of nonfat milk powder at room temperature for 30 min. Finally, the bands recognized by the specific antibody were visualized using a chemiluminescence-based ECL system (Amersham Biosciences) and exposed to an X-ray film for 30 min (Eastman Kodak). A computer imaging system (Gel-Pro Analyzer) was utilized to measure the intensity of the OD of the bands.

#### 2.4.6. Evaluation of Nitrite Levels after Treatment with EEGP and Bioactive Fraction

The mice were pretreated with EEGP or aqueous fraction (10 mg/kg, s.c.). The negative control group received the vehicle. After 30 min of treatment, it was applied carrageenan IP at a dose of 500 *μ*g/cavity. After 4 h, the animals were killed and the peritoneal cavity washed with 3 mL of PBS/EDTA (1 mM). The production of NO was determined in the peritoneal lavage using the Griess method [22] by measuring the nitrite concentration at 540 nm.

#### 2.4.7. Evaluation of the Activity of EEGP and Its Bioactive Fraction on Carrageenan-Induced Paw Edema

The mice were pretreated with EEGP or aqueous fraction (1, 3, 10, or 30 mg/kg, IP). The negative control group received the vehicle. After 30 min of treatment, the animals were subjected to an intraplantar injection of 50 *μ*L carrageenan (1 mg/paw) in the left hind leg. The animal's paw volume was measured before (time 0) and after injection of carrageenan (1, 2, 3, 4, and 5 h) utilizing a plethysmometer (Ugo Basile, Model 7150, Italy). The results were expressed as the threshold of the inflamed paw Δ (mL), which was calculated by subtracting the values obtained before (time 0) and after the injection of carrageenan [23].

### 2.5. Chemical Analysis of the Bioactive Fraction

#### 2.5.1. High-Performance Liquid Chromatography Reverse Phase

Ten microliters of sample (aqueous fraction) were injected into a liquid chromatograph coupled to a photodiode array detector at 260 nm, and the sample were eluted through a C18 reverse phase column (250 × 4.6 mm), with particle size of 5 *μ*m. The mobile phase was water/acetic acid (98/2) (solvent A) and water/acetonitrile/acetic acid (68/30/2) (solvent B) with constant rate of 1 mL/min. The gradient started with 0 to 30% of solvent B in 20 min, 30 to 50% of B in 10 min, 50 to 70% of B in 20 min, 70 to 100% of B in 5 min, 100% of B in 20 min, and 100 to 0% of B in 10 min [24]. We utilized authentic standards of flavonoids and phenolic acids to compare the retention time of substances in the aqueous fraction sample.

### 2.6. Statistical Analysis

Data were expressed as mean ± standard error of the mean (SEM), and statistical comparisons between groups were made utilizing analysis of variance (ANOVA) followed by Tukey test. Significance was accepted when *P* < 0.05.

## 3. Results

### 3.1. EEGP and Aqueous Fraction Inhibit Neutrophil Migration in the Peritoneal Cavity

**Figure 1:**
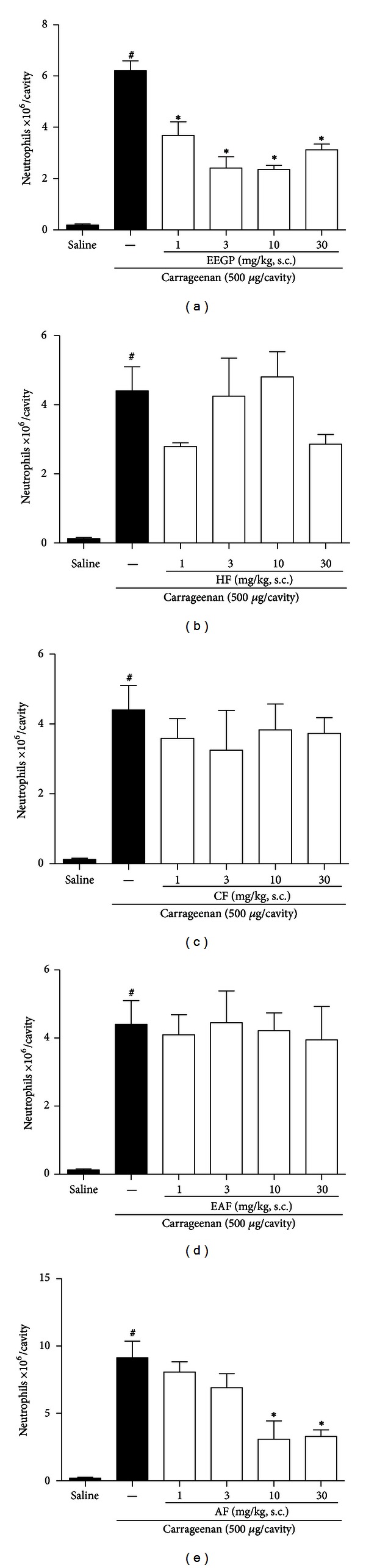
Inhibitory effect of the ethanolic extract of geopropolis (EEGP) and aqueous fraction (AF) on neutrophils migration into the peritoneal cavity induced by carrageenan. Neutrophil migration was determined 4 h after the injection of carrageenan 500 *μ*g/cavity. Mice previously treated with vehicle (saline and carrageenan), EEGP (a), hexane fraction (HF-(b)), chloroform (CF-(c)), ethyl acetate (EAF-(d)), or aqueous (AF-(e)). The data are expressed as mean ± SEM, *n* = 6. Symbols indicate statistical difference (*P* < 0.05, Tukey test). ^#^Compared to the saline group; *compared to the carrageenan group.

We evaluated the activity of EEGP and its aqueous fraction on neutrophil migration in the peritoneal cavity induced by carrageenan. Regarding the results, it was found that administration of EEGP decreased the influx of neutrophils into the peritoneal cavity, compared to the carrageenan group (*P* < 0.05). It was observed an inhibition of 41, 61, 62, and 50% for doses of 1, 3, 10, and 30 mg/kg (Figure 1(a)), respectively. Concerning the chemical fractions studied, the hexane fraction (Figure 1(b)), the chloroform fraction (Figure 1(c)), and the ethyl acetate fraction (Figure 1(d)) showed no significant inhibition of neutrophil recruitment (*P* > 0.05). On the other hand, the aqueous fraction decreased the number of neutrophils in the peritoneal cavity after injection of carrageenan (*P* < 0.05), where we observed an inhibition of 66 and 64% for doses of 10 and 30 mg/kg, respectively (Figure 1(e)). Thus, the aqueous fraction was selected as the bioactive fraction of EEGP.

### 3.2. EEGP and Aqueous Fraction Inhibit the Rolling and Adhesion of Leukocytes

**Figure 2:**
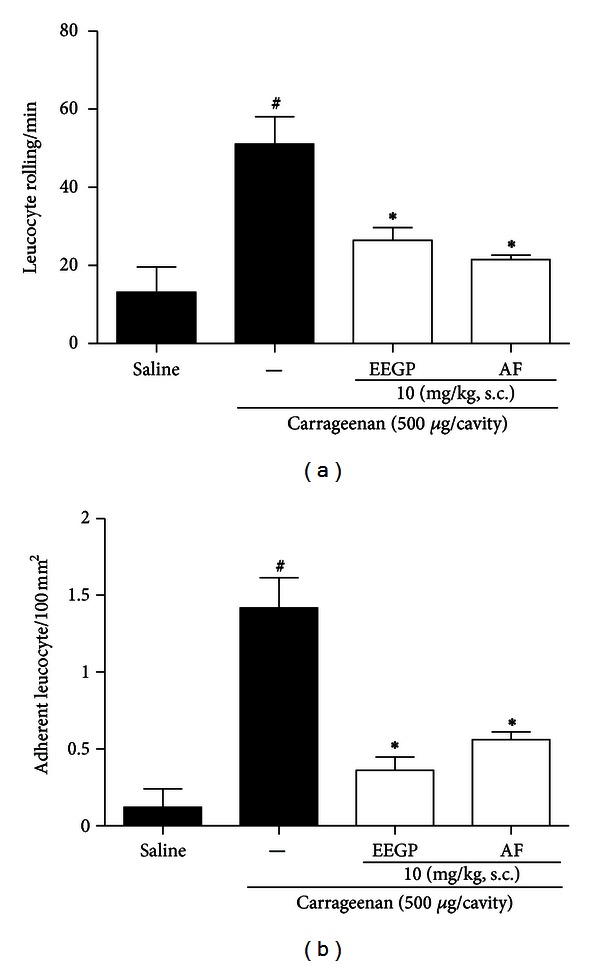
Inhibitory effect of the ethanolic extract of geopropolis (EEGP) and aqueous fraction (AF) on rolling (a) and adhesion (b) of leukocytes assessed by intravital microscopy in mesenteric tissue of mice, 2 and 4 h after IP injection of carrageenan 500 *μ*g/cavity. Mice were pretreated with vehicle (saline and carrageenan), EEGP, or aqueous fraction (10 mg/kg). The data are expressed as mean ± SEM, *n* = 5. Symbols indicate statistical difference (*P* < 0.05, Tukey test). ^#^Compared to the saline group; *compared to the carrageenan group.

By checking the activity on the inhibition of neutrophil migration in the peritoneal cavity, we evaluated the activity of EEGP and aqueous fraction on the rolling and adhesion of leukocytes to endothelial cells. Based on the results, we found that EEGP and the aqueous fraction, at a dose of 10 mg/kg, decreased leukocyte rolling and adhesion to endothelial cells, compared to the carrageenan group (*P* < 0.05), with an inhibition of 48 and 58% for the rolling (Figure 2(a)) and 75 and 61% for the adhesion of leukocytes (Figure 2(b)), respectively.

### 3.3. EEGP and Aqueous Fraction Promote No Changes Chemokines Levels in Mice Subjected to Injection of Carrageenan in the Peritoneal Cavity

**Figure 3:**
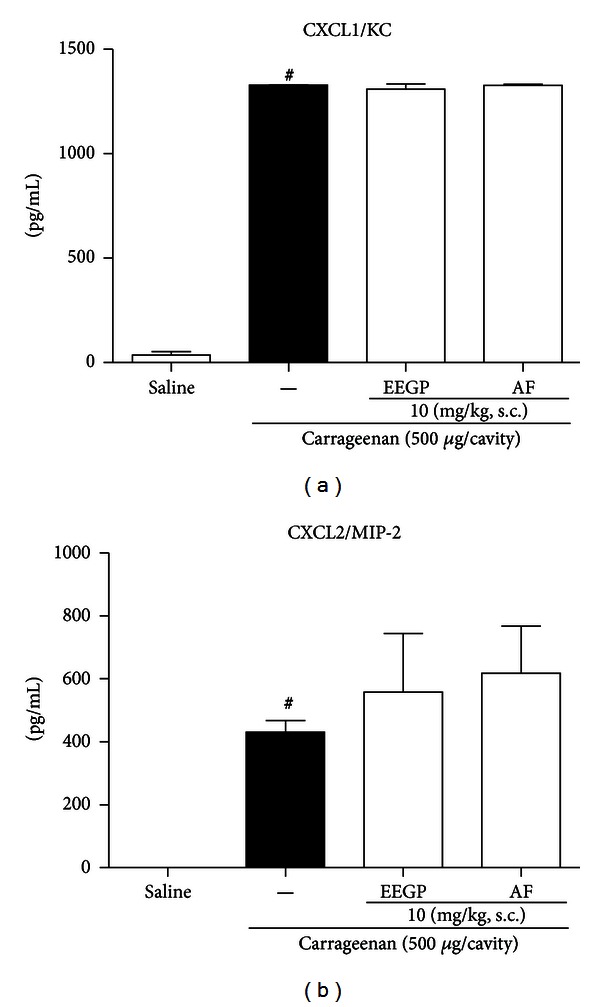
Effect of the ethanolic extract of geopropolis (EEGP) and aqueous fraction (AF) on the levels of CXCL1/KC (a) and CXCL2/MIP-2 (b) 3 h after the injection of 500 *μ*g/cavity of carrageenan in the peritoneal cavity. Mice were pretreated with vehicle (saline and carrageenan), EEGP, or aqueous fraction (10 mg/kg, s.c.). The data are expressed as mean ± SEM, *n* = 5. Symbols indicate statistical difference (*P* < 0.05, Tukey test). ^#^Compared to the saline group.

In order to elucidate the mechanism of action by which EEGP and the aqueous fraction inhibit neutrophil migration, we initially studied their action on the synthesis and/or release of chemokines. Thus, we found that EEGP and the aqueous fraction, at a dose of 10 mg/kg, did not alter levels of chemokines CXCL1/KC (Figure 3(a)) and CXCL2/MIP-2 (Figure 3(b)) in animals subjected to IP injection of carrageenan (*P* > 0.05).

### 3.4. Inhibitors of NO Pathway Cause Suppression of the Effect of EEGP and Aqueous Fraction on Neutrophil Migration Induced by Carrageenan

**Figure 4:**
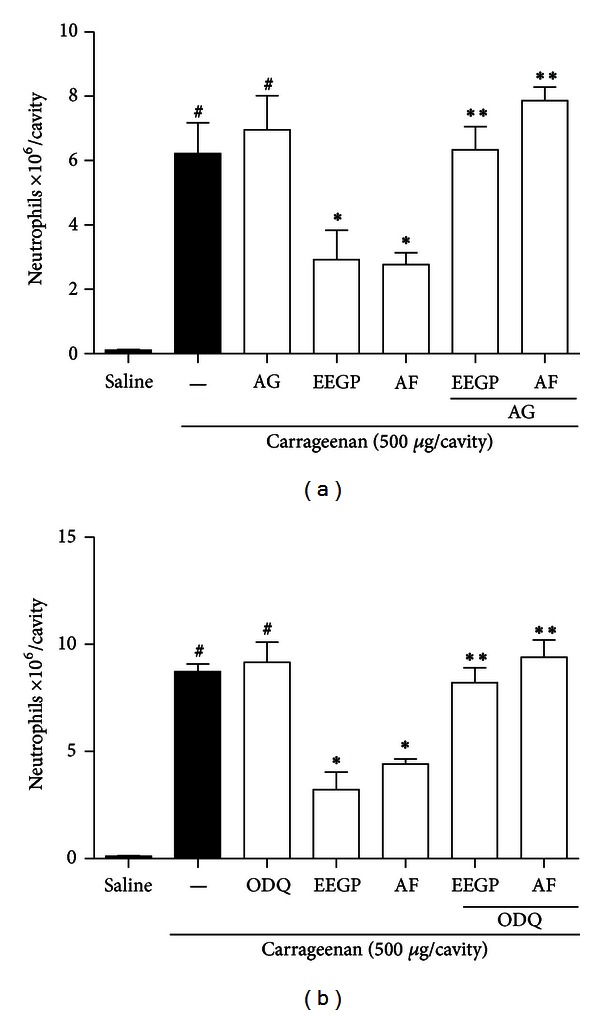
Inhibitory effect of the ethanolic extract of geopropolis (EEGP) and aqueous fraction (AF) on neutrophils migration into the peritoneal cavity induced by carrageenan. Neutrophil migration was determined 4 h after the injection of carrageenan 500 *μ*g/cavity. Mice were pretreated with aminoguanidine (AG—50 mg/kg, (a)) and ODQ (5 *μ*mol/kg, (b)) antagonists. After 30 min, the animals were pretreated with vehicle (saline and carrageenan), EEGP, or aqueous fraction (10 mg/kg). The data are expressed as mean ± SEM, *n* = 6. Symbols indicate statistical difference (*P* < 0.05, Tukey test). ^#^Compared to the saline group; *compared to the carrageenan group; **compared to the groups that received only EEGP and the aqueous fraction.

After, we evaluated the participation of the NO pathway. According to the results, we observed that the application of antagonists of NO pathway (Figures 4(a) and 4(b)) abolished the inhibitory effect of EEGP and aqueous fraction, at a dose of 10 mg/kg, on neutrophil migration induced by carrageenan (*P* < 0.05), thus confirming that the inhibitory action of recruitment of neutrophils is at least partly due to the NO pathway.

### 3.5. EEGP and Aqueous Fraction Decrease the Expression of ICAM-1 and Increase Nitrite Levels in Mice Subjected to Injection of Carrageenan in the Peritoneal Cavity

**Figure 5:**
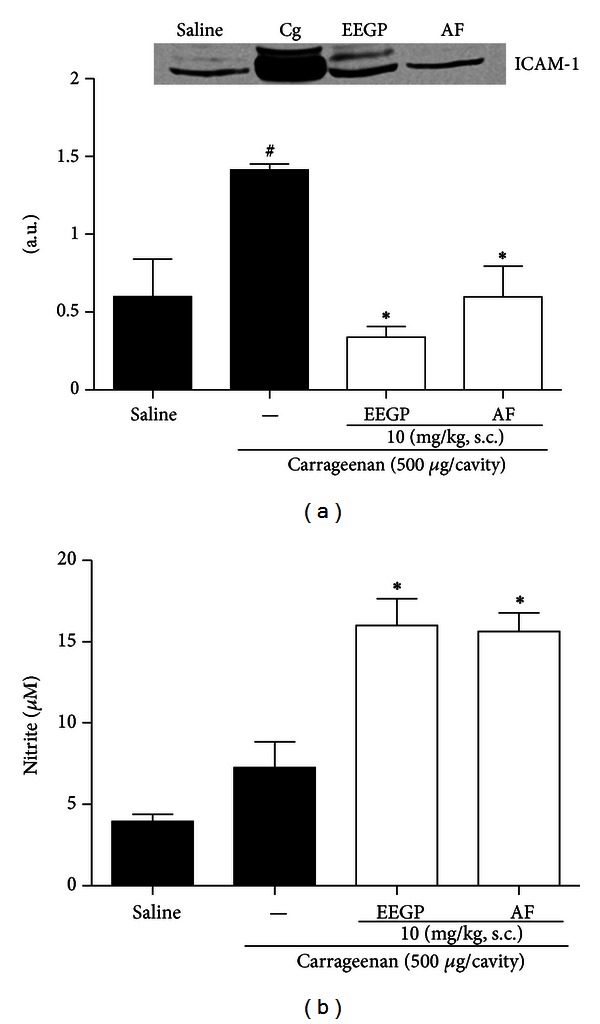
Effect of the ethanolic extract of geopropolis (EEGP) and aqueous fraction (AF) on the expression of ICAM-1 (a) and nitrite levels (b) 4 h after the injection of 500 *μ*g/cavity of carrageenan (Cg) in the peritoneal cavity. Mice were pretreated with vehicle (saline and carrageenan), EEGP, or aqueous fraction (10 mg/kg, s.c.). The data are expressed as mean ± SEM, *n* = 5. Symbols indicate statistical difference (*P* < 0.05, Tukey test). ^#^Compared to the saline group; *compared to the carrageenan group.

Regarding the expression of adhesion molecules ICAM-1, we observed that treatment with EEGP and aqueous fraction, at a dose of 10 mg/kg, reduced its expression (*P* < 0.05) when compared to the carrageenan group (Figure 5(a)). Besides, the treatment with EEGP and aqueous fraction (10 mg/kg) increased the nitrite levels (*P* < 0.05) when compared to the carrageenan group (Figure 5(b)).

### 3.6. Antiedematogenic Activity of EEGP and Aqueous Fraction on Paw Edema

We verified the activity of EEGP and aqueous fraction on carrageenan-induced paw edema. According to the results, we found that EEGP showed antiedematogenic activity (Table 1), and we observed an inhibition of 57, 60, 62, and 62% for doses of 1, 3, 10, and 30 mg/kg (3 h) and 66% (4 and 5 h after injection of carrageenan) for the 30 mg/kg dose.

Furthermore, the aqueous fraction was able to reduce the edema only for the dose of 30 mg/kg, showing an inhibition of 56% (3 h) and 49 % (4 h) (Table 2).

### 3.7. High-Performance Liquid Chromatography Reverse Phase

**Figure 6:**
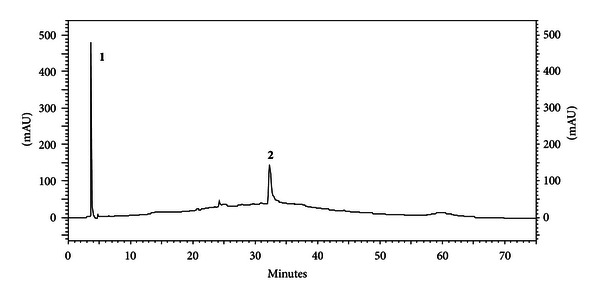
Chromatograms obtained by HPLC-RP of the aqueous fraction. **1**: UV *λ* 240 nm, RT = 3.71 min; **2**: UV *λ* 238 nm, RT = 32.36 min.

According to the chromatograms obtained by high-performance liquid chromatography reverse phase (HPLC-FR) of the aqueous fraction (Figure 6), we could verify the existence of two major compounds that were not compatible with any of the standards utilized.

## 4. Discussion

The present study demonstrated that the EEGP and its aqueous fraction decreased leukocyte interaction (rolling and adhesion) with endothelial cells as well as neutrophil migration into the peritoneal cavity of mice subjected to intraperitoneal injection of carrageenan through suppression of adhesion molecules ICAM-1, which is an NO pathway-dependent activity.

The inflammatory process is a set of events arising from the participation of various mediators that promote vascular and cellular events [5, 25]. NO plays different physiological functions in the body. Its production involves the participation of a family of enzymes known as NO synthases (NOS). Among them, the inducible NOS (iNOS) is activated during the inflammatory process [18, 21, 26].

Recent studies have shown that the inhibitory effect of NO (via iNOS) on migration of neutrophils in the inflammatory process is dependent on the activation of enzyme soluble guanylate cyclase (sGC). The activation of the sGC leads to an increase in the production of guanosine 3′5′-cyclic monophosphate (cGMP) and consequent inhibition of the ICAM-1 adhesion molecules [18, 21].

The present study demonstrated that treatment with specific antagonists of iNOS or the sGC suppressed the inhibition of neutrophil migration by EEGP and its aqueous fraction. These results suggest, therefore, that the inhibitory effect of EEGP and the aqueous fraction on neutrophils migration by NO pathway is related to increased levels of NO (via iNOS activation), which can be observed by the increase in nitrite levels and consequent activation of the sGC/cGMP pathway, thus leading to the suppression of ICAM-1 adhesion molecules.

As edema is one of the key events in the inflammatory response, we also evaluated the effectiveness of EEGP and the aqueous fraction on carrageenan-induced paw edema. The first phase (0–3 h) of carrageenan-induced paw edema is associated with increase in the histamine, serotonin, and kinins chemical mediators, and these are related with the increase in vascular permeability, besides the increased production of the IL-1*β* and TNF-*α* cytokines [27–29]. In the second phase (3-4 h) there is a production of mediators derived from the inducible cyclooxygenase enzyme, such as prostaglandins, and this second phase is sensitive to anti-inflammatory cyclooxygenase inhibitors [27, 28]. According to the results presented, EEGP and its aqueous fraction decreased the carrageenan-induced paw edema, where this activity was observed only in the second phase (3-4 h) of the experiment. These results, therefore, suggest that the activity of EEGP and the aqueous fraction may be related to the inhibition of prostaglandin formation.

In relation to chemical analysis by HPLC, with mobile phase optimized for polar compounds, two major compounds could be identified, which shows the highly polar characteristic of bioactive chemical compounds. No flavonoid or phenolic acid was found in the aqueous fraction. These phenolics are a class of chemicals reported in the literature as responsible for most of the biological activity of propolis from *Apis mellifera* [30, 31], thus suggesting a different chemical profile of the geopropolis from *Melipona scutellaris*, as evidenced in the aqueous fraction. Hence, these chemical profile data suggest the presence of chemical compounds that have not been reported in the literature as to their pharmacological activities (especially anti-inflammatory), which arouses special attention due to its promising mechanism of action.

## 5. Conclusion

Therefore, we conclude that the EEGP and its aqueous fraction decreased migration of neutrophils in the inflammatory process, and this is dependent on the nitric oxide pathway. Thus, due to their distinct chemical profile and promising mechanism of action, further studies are necessary for the isolation and identification of bioactive compounds present in the aqueous fraction, which may enable the development of new effective anti-inflammatory drugs for the treatment of inflammatory diseases. 

## Figures and Tables

**Table 1:** EEGP effect on carrageenan-induced paw edema on mice.

Treatment (mg/kg)	Time (h) after injection of carrageenan				
	1	2	3	4	5
Carrageenan	0.06 ± 0.01	0.08 ± 0.01	0.13 ± 0.02	0.16 ± 0.01	0.15 ± 0.01
EEGP 1 mg/kg	0.04 ± 0.01	0.05 ± 0.02	0.06 ± 0.02*	0.11 ± 0.02	0.12 ± 0.02
EEGP 3 mg/kg	0.05 ± 0.01	0.06 ± 0.01	0.05 ± 0.01*	0.12 ± 0.01	0.15 ± 0.01
EEGP 10 mg/kg	0.05 ± 0.01	0.05 ± 0.01	0.05 ± 0.02*	0.12 ± 0.01	0.13 ± 0.01
EEGP 30 mg/kg	0.05 ± 0.01	0.05 ± 0.01	0.05 ± 0.01*	0.06 ± 0.01*	0.05 ± 0.02*

The data are expressed as mean ± SEM, *n* = 6. Symbols indicate statistical difference (*P* < 0.05, Tukey test). *Compared to the carrageenan group.

**Table 2:** Aqueous fraction effect on carrageenan-induced paw edema on mice.

Treatment (mg/kg)	Time (h) after injection of carrageenan				
	1	2	3	4	5
Carrageenan	0.08 ± 0.01	0.10 ± 0.02	0.13 ± 0.01	0.16 ± 0.02	0.15 ± 0.01
Aqueous fraction 1 mg/kg	0.10 ± 0.01	0.11 ± 0.01	0.11 ± 0.01	0.14 ± 0.01	0.13 ± 0.01
Aqueous fraction 3 mg/kg	0.08 ± 0.01	0.09 ± 0.02	0.09 ± 0.01	0.13 ± 0.01	0.13 ± 0.01
Aqueous fraction 10 mg/kg	0.06 ± 0.01	0.08 ± 0.01	0.08 ± 0.02	0.11 ± 0.02	0.11 ± 0.01
Aqueous fraction 30 mg/kg	0.06 ± 0.01	0.05 ± 0.02	0.06 ± 0.02*	0.08 ± 0.03*	0.09 ± 0.02

The data are expressed as mean ± SEM, *n* = 6. Symbols indicate statistical difference (*P* < 0.05, Tukey test). *Compared to the carrageenan group.

## Abbreviations

AG: Aminoguanidine
cGMP: Guanosine 3′5′-cyclic monophosphate
DMSO: Dimethylsulphoxide
EEGP: Ethanolic extract of geopropolis
HPLC-FR: High-performance liquid chromatography reverse phase
ICAM-1: Intercellular adhesion molecule type 1
iNOS: Inducible nitric oxide synthase
NO: Nitric oxide
NOS: Nitric oxide synthases
sGC: Soluble guanylate cyclase
ODQ: [1H-(1,2,4)oxadiazolo (4,3-a) quinoxalin-1-one].
